# Respiratory viral infections in lung transplantation: Recent advances in epidemiology, clinical impact, and therapeutic approaches

**DOI:** 10.1016/j.jhlto.2025.100362

**Published:** 2025-08-05

**Authors:** Hannah Bahakel, Lara Danziger-Isakov

**Affiliations:** Department of Pediatrics, University of Cincinnati College of Medicine, Cincinnati Children’s Hospital Medical Center, Cincinnati, OH

**Keywords:** Lung transplantation, Respiratory viral infections, Influenza, Respiratory syncytial virus, Vaccination

## Abstract

Respiratory viruses encompass a diverse group of viruses, including influenza, respiratory syncytial virus (RSV), parainfluenza (PIV), human metapneumovirus (hMPV), severe acute respiratory syndrome coronavirus 2 (SARS-CoV-2), and adenovirus. Lung transplant recipients are particularly vulnerable to complications from respiratory viral infections (RVIs), leading to increased morbidity and mortality. This heightened risk is a result of both anatomical and functional modifications from transplant surgery, as well as immunosuppressive therapy. Beyond the immediate morbidity associated with infection, RVIs are also recognized for their association with both acute rejection and chronic lung allograft dysfunction (CLAD)/bronchiolitis obliterans syndrome (BOS). This article provides updated insights into epidemiology, clinical outcomes, prevention strategies, and treatment options for RVIs in lung transplant recipients.

## Introduction

**Figure 1:**
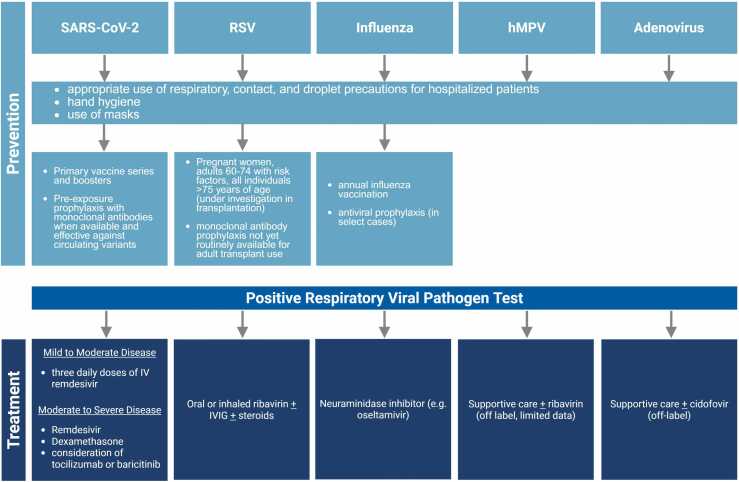
Insights into epidemiology, clinical outcomes, prevention strategies, and treatment options for RVIs in lung transplant recipients.

Respiratory viruses encompass a diverse group of viruses, including influenza, respiratory syncytial virus (RSV), parainfluenza (PIV), human metapneumovirus (hMPV), severe acute respiratory syndrome coronavirus 2 (SARS-CoV-2), and adenovirus. Lung transplant recipients are particularly vulnerable to complications from respiratory viral infections (RVIs), leading to increased morbidity and mortality. This heightened risk is a result of both anatomical and functional modifications from transplant surgery, as well as immunosuppressive therapy. Beyond the immediate morbidity associated with infection, RVIs are also recognized for their association with both acute rejection and chronic lung allograft dysfunction (CLAD)/bronchiolitis obliterans syndrome (BOS). While most respiratory viruses are acquired post-transplant, there is increasing recognition that donor derived transmission can occur, sometimes resulting in severe complications. Donor-derived infections will be addressed further in a separate article, but the potential for donor-derived respiratory viral infections in lung transplant recipients underscores the ongoing challenge of optimizing preventive and therapeutic strategies in this high-risk population. This article provides updated insights into epidemiology, clinical outcomes, prevention strategies, and treatment options for RVIs in lung transplant recipients (Figure 1).

## Critical analysis of existing literature

### SARS-CoV-2

#### Epidemiology and outcomes

The emergence of SARS-CoV-2 and coronavirus disease (COVID-19) has had profound impact on lung transplant recipients, exposing them to severe respiratory disease, characterized by tachypnea or hypoxia, increased morbidity and mortality, and disruptions in transplant care. While the prototype SARS-CoV-2 virus was first sequenced in January of 2020, as the pandemic evolved, the ancestral virus strain underwent multiple spike protein mutations, resulting in the emergence of several variants, including Alpha, Beta, Gamma, Delta, Epsilon, and Omicron. The clinical trajectory of COVID-19 in lung transplant recipients has evolved substantially throughout the course of the pandemic, shaped by the emergence of new variants, the advancement of preventative and management strategies, and the widespread adoption of vaccination, resulting in changes to incidence rates and outcomes over time (Table 1). Accumulating evidence from large longitudinal cohorts indicates that the severity of COVID-19 in solid organ transplant recipients has decreased over successive SARS-CoV-2 waves, largely attributable to the emergence of less virulent variants, increased vaccine uptake, and improved access to early antiviral therapies.^1^ Early in the pandemic, during the Wildtype, Alpha, and Delta waves, lung transplant recipients experienced high rates of severe COVID-19, hospitalization, and mortality. In a comprehensive cohort study of 1975 solid organ transplant recipients, including 417 lung transplant recipients, spanning March 2020 to May 2023, Solera et al. demonstrated a marked reduction in severe COVID-19 from 44.6% during the Wildtype/Alpha/Delta period to 5.7–16.1% during Omicron waves. Lung transplantation was identified as the strongest organ-specific risk factor for severe COVID-19 (OR: 4.62, 95% CI: 2.1–7.89).^1^ This finding is corroborated by multiple large-scale studies. Hall et al. reported a 31.3% mortality rate among lung transplant recipients with COVID-19 compared with recipients of other organ transplants, including kidney (14.8%), heart (4.4%), liver (11.4%), and kidney-pancreas (12.0%). Furthermore, a large multicenter cohort study conducted in 1616 adult solid organ transplant recipients early in the pandemic described an increase in 28-day mortality in lung transplant recipients compared to recipients of other solid organs, (24% vs 16%, respectively).^2^ Hospitalization occurred in a higher proportion of lung recipients (75%) compared to non-lung solid organ transplant recipients (66%). Lung transplant was independently associated with death after adjusting for age and comorbidities. Risk factors for mortality and severe COVID-19 in this population include CLAD, defined as a persistent decreased in forced expiratory volume in 1 s (FEV_1_) of more than 20% from baseline with no reversible causes identified,^3^ older age, and higher burden of comorbidities.

**Table 1:** Outcomes of COVID-19 Variants in Lung Transplant Recipients

Variant/Wave	Hospitalization Rate	ICU Admission/Severe Disease	Mortality Rate	Key Findings in Lung Transplant Recipients	References
Wildtype/Alpha/Delta	High (up to 58.5%)	Higher than Omicron; Delta was highest of all waves	High (up to 24.1%)	• Highest severity • Lung transplant recipients at greatest risk for severe outcomes. ICU admission and death more common with Delta wave than with Omicron • prolonged hospital stays	1, 20, 32, 99, 100, 101
Omicron (BA.1, BA.2, BA.5)	Lower (18.4−30%)	Lower than Delta	Lower (2−6.4%)	Reduced severity, but LTRs still at higher risk than other SOT; long hospital stays	102, 103, 104, 105
Omicron (XBB.1.5, BQ.1.1)	Low	Low	Low	Hospitalization rates remain higher in LTRs vs. other SOT	^1^

In the general population, morbidity and mortality changed in relation to the circulating SARS-CoV-2 variant, with lower hospitalization and mortality rates during the Omicron period as compared to the earlier Delta period, which appeared to be particularly virulent.^4^, ^5^ While a marked reduction in COVID-19 severity was observed across solid organ transplant recipients, including lung transplant recipients during the Omicron wave, cohort studies performed during this wave suggested that lung transplant recipients with COVID-19 continue to have high hospitalization rates and prolonged hospital stays despite the reduced virulence of the Omicron variant.

Limited data are available regarding lung transplant function following COVID-19. One report of 74 lung transplant patients documented long-term adverse effects on the allograft following COVID-19, with a significant decline in spirometry for several months.^6^ Lung function gradually improved by six months post-infection but remained significantly lower than pre-COVID-19 levels.

Several publications have attempted to delineate the association between COVID-19 and increased preexisting or *de novo* human leukocyte antigen (HLA) donor specific antibodies (DSA). A retrospective study of 24 patients diagnosed with SARS-CoV-2 infection were tested for the presence of HLA class I and II DSA pre-COVID-19 and at 1,3, and 6 months post-COVID-19 diagnosis. No significant differences in the levels of calculated panel of reactive antibodies (cPRA) or HLA class I and II antibodies were observed post-COVID-19 diagnoses compared to pre-COVID-19, suggesting no significant association between SARS-CoV-2 infection on pre-existing or *de novo* DSA.^7^ Alternatively, a similar retrospective study of 63 lung transplant recipients diagnosed with COVID-19 described an association between the severity of SARS-CoV-2 infection and pre-existing or *de novo* DSA.^8^ De-novo DSA was detected post-COVID-19 diagnosis in none of those with mild disease, 2.4% in those with moderate disease, and 33.3% in those with severe COVID-19. In this cohort, mild to moderate COVID-19 was not associated with increased preexisting or *de novo* DSA, consistent with findings from the previously mentioned smaller cohort. However, the increase in DSA in those with severe disease led to the postulation that severe SARS-CoV-2 infection may be associated with increased risk of *de novo* DSA production resulting in allograft dysfunction. Notably, the large number of lung transplant recipients that died from severe COVID-19 respiratory complications (16/25) prior to HLA antibody testing in this study may not have permitted the calculation of a significant correlation between severe COVID and DSA production.

Although it is well established that lung transplant recipients experience increased morbidity and mortality following SARS-CoV-2 infection, the long-term sequelae, including alloimmunity and development of CLAD, are still being elucidated.

#### Prevention

Efforts to prevent progression to severe COVD-19 in lung transplant recipients with pre-exposure prophylaxis with the monoclonal antibodies tixagevimab and cilgavimab have yielded disappointing results. Breakthrough of COVID-19 was common despite prophylaxis, perhaps due to rapid variant evolution and diminished efficacy of monoclonal antibodies against prevalent circulating variants.^9^, ^10^ Additional monoclonal antibodies targeting emerging variants have become commercially available; however, data on their use in lung transplant recipients remain limited. Pemivibart currently has emergency use authorization for pre-exposure prophylaxis in lung transplant recipients based on the results of in vitro neutralization assays.^11^ Evidence on its efficacy in the lung transplant setting is lacking, and its effectiveness against future circulating SARS-CoV-2 variants remains uncertain.

Given the limitations of pre-exposure prophylaxis in effectively preventing COVID-19 among lung transplant recipients, vaccination remains a critical strategy in reducing disease severity and improving outcomes in this high-risk population. Lung transplant recipients experienced reduced humoral and cellular immune responses after two doses of COVID-19 vaccination when compared to healthy controls, with only 28–56% of lung transplant recipients developing detectable anti-spike IgG after two doses and up to 55% after a third dose. Cellular responses are also diminished; a systematic review and meta-analysis of the immunogenicity of COVID-19 vaccines in solid organ transplant recipients reported that the overall proportion of lung transplant recipients who developed a cellular immune response after two vaccine doses was 56.9% (95% CI 14.5–99.2%).^12^, ^13^, ^14^, ^15^ Three doses of mRNA vaccine have been shown to elicit enhanced immunogenicity with improved rates of serum neutralizing antibodies against SARS-CoV-2 variants versus a 2-dose vaccine series.^16^, ^17^ Randomized and observational studies demonstrate that the third dose increases the proportion of serological responders to approximately 54–77%, and also improves the breadth of neutralization against circulating variants, with a significant proportion of previously non-responding patients achieving seroconversion and measurable neutralizing activity after the booster. In a secondary analysis of a randomized trial, the proportion of transplant recipients with positive neutralization against the Delta variant increased from 18% after two doses to 55% after a third dose.^17^ Ferreria et al. also demonstrated that lung transplant recipients and other solid organ transplant recipients, even after partial or full vaccination, generally mount weaker neutralizing antibody and T-cell responses to the Omicron BA.1 and other variants compared to health controls. However, those with prior infection plus vaccination (hybrid immunity) achieved the most robust BA.1 specific and cross-neutralizing responses, approaching those of triple-vaccinated immunocompetent controls, indicating that infection can significantly boost both homotypic and heterotypic immunity in this population.^18^

Despite the dampened immunogenicity of vaccination, a clinical effectiveness study conducted in adult solid organ transplant recipients that included 205 lung transplant recipients demonstrated an almost 80% reduction in the incidence of symptomatic COVID-19 compared to unvaccinated transplant recipients (incidence rate was 0.065 per 1000 /person days in the vaccinated group versus 0.34 per 1000/person days in the control group).^19^ Based on these data, current guidelines recommend lung transplant recipients to complete a three dose primary series of the COVID-19 vaccine, followed by two booster doses spaced six months apart.

#### Treatment

Management of COVID-19 in lung transplant recipients typically involves a combination of antiviral therapy, immunomodulation, and supportive care. Remdesivir, a nucleotide inhibitor of the SARS-CoV-2 RNA polymerase, remains the primary anti-viral agent for early treatment, with studies suggesting that it is safe and may reduce progression to severe disease in transplant recipients.^20^, ^21^ An observational study of 192 solid organ transplant recipients, including 37 lung transplant recipients, indicated that early outpatient treatment with remdesivir significantly decreased the hospitalization rate with an adjusted hazard ratio of 0.12.^22^ The number needed to treat with remdesivir to prevent one hospitalization was 15.2. Similarly, a recent retrospective cohort of 130 lung and heart-lung transplant recipients suggested a potential long-term benefit of remdesivir in preserving lung function in lung transplant recipients, with a notable decline in FEV1at 3 months post-infection in those not treated with remdesivir.^21^ Molnupiravir is an oral antiviral agent developed for mild to moderate COVID-19 in non-hospitalized adults at high risk for disease progression. While a retrospective cohort of 113 lung transplant recipients reported similar rates of hospitalization, ICU admission, and mortality as compared to those treated with remdesivir, molnupiravir is not recommended for treatment in lung transplant recipients due to risk of inducing viral mutation and the paucity of data regarding its clinical efficacy.^23^, ^24^ The use of nirmatrelvir-ritonivir, while highly effective in preventing severe disease and hospitalization, is limited in transplant patients due to significant drug-drug interactions, particularly with calcineurin inhibitors.^24^, ^25^ Monoclonal antibodies (mAbs), such as sotrovimab, bebtelovimab, and casirivimab/imdevimab have been used under emergency use authorization, though their utility diminished with the emergence of both in vitro neutralization data demonstrating resistance against circulating SARS-CoV-2 variants and clinical evidence of treatment-emergent resistance mutations.^26^, ^27^

The role of corticosteroids and immunomodulatory agents in the management of COVID-19 among lung transplant recipients has been primarily informed by studies in the general population, with limited transplant-specific data. Corticosteroids, particularly dexamethasone, are recommended for patients requiring supplemental oxygen or ventilatory support, and have become standard of care in this context, including among transplant recipients.^28^ In more severe cases, adjunctive immunomodulatory therapies such as interleukin-6 (IL-6) receptor antagonists (e.g., tocilizumab) or Janus kinase (JAK) inhibitors (e.g., baricitinib) have been utilized to prevent development of respiratory distress syndrome induced by COVID-19 related cytokine release syndrome based on evidence demonstrating improved clinical outcomes in select populations. Retrospective data suggest that the use of tocilizumab and baricitinib is well tolerated with a minimal risk of secondary infections.^29^, ^30^, ^31^, ^32^ However, robust efficacy data are lacking. A study among 228 immunocompromised patients hospitalized with severe COVID-19 in six Israeli hospitals reported that baricitinib and tocilizumab therapy was not associated with reduced mortality compared to standard of care.^33^ Larger prospective studies with well-matched control groups are needed to better address efficacy of these agents in the transplant setting.

### RSV

#### Epidemiology and outcomes

RSV has become increasingly recognized as a concerning respiratory pathogen in lung transplant recipients. Morbidity is especially pronounced in lung transplant recipients compared to other solid organ transplant recipients.^34^, ^35^, ^36^ RSV has an incidence of approximately 2–16% in adult lung transplant recipients and appears to cause lower respiratory tract disease more frequently compared to other community acquire respiratory viruses.^37^ In a 9-year retrospective multicenter cohort study that included adult lung transplant recipients with confirmed RSV infection, approximately 30% developed lower respiratory tract involvement.^38^ Risk factors for progression to lower respiratory tract disease and mortality in this population remain poorly defined but include younger age (children <2 years old), recent transplantation, and recent treatment for rejection. Mortality rates ranging from 10–20% have been observed even with receipt of appropriate treatment and supportive care; although more recent studies suggest that attributable mortality is much lower.^34^, ^38^, ^39^

Outcomes of lung transplant grafts following RSV infection remain inconsistent across studies. The presence of lower respiratory tract involvement with RSV may significantly increase the subsequent risk of allograft dysfunction. A systematic review and meta-analysis published in 2021 demonstrated that progression to CLAD 180–360 days postinfection was substantial (pooled incidences 19–24%) and associated with severe infection.^39^ Similarly, the previously mentioned retrospective multicenter cohort study showed that 28.8% of recipients developed new allograft dysfunction 3 months after infection.^38^

#### Prevention

Mitigating the burden of RSV infection in transplant recipients has historically depended upon prevention of transmission, including contact isolation for hospitalized patients with suspected or confirmed RSV.^40^ However, additional strategies for the prevention of RSV illness in high-risk populations have recently emerged. The Food and Drug Administration (FDA) has approved two recombinant subunit RSV vaccines and one mRNA vaccine for the prevention of RSV-associated lower respiratory tract disease. In randomized controlled trials performed in pregnant women and individuals >60 years of age, all three vaccines demonstrated efficacy (74.5–84.4%) in preventing RSV-associated lower respiratory tract disease.^41^, ^42^, ^43^, ^44^, ^45^ Current licensure is limited to pregnant women, adults ages 60–74 who are at increased risk of severe RSV, and all individuals over the age of 75. Immunocompromised individuals, including solid organ transplant recipients, were excluded from these trials, so efficacy and safety data in these populations are limited. Clinical trials are currently underway to evaluate the safety and immunogenicity of RSV vaccines in adult solid organ transplant recipients (Table 2, ClinicalTrials.gov NCT06067230).

**Table 2:** Completed or Ongoing Clinical Trials of Respiratory Virus Vaccines and Therapeutics in Transplant Recipients

Virus	Intervention	Trial Phase and Design	Study Population	Outcome Measures	Key Findings	Trial registry/Citation
RSV	Presatovir	Phase 2b, RCT, double-blind, placebo-controlled	LTRs with RSV	Change in nasal RSV load, symptoms, lung function	No significant improvement in viral load, symptoms, or lung function	NCT02534350^58^
	Prefusion F protein vaccine	Phase 3, randomized, double-blind, placebo-controlled	SOT recipient (kidney, liver, lung, or heart)	Safety, tolerability, and immunogenicity	Enrollment completed, results not yet available	NCT05842967
	mRNA−1345 vaccine	Phase 3, randomized, Part A: double-blind, Part B: open-label	SOT recipient (kidney, liver, lung)	Safety and immunogenicity	Active, not recruiting	NCT06067230
Influenza	High-dose IIV, MF59-adjuvanted IIV compared to standard dose IIV (STOP-FLU)	Phase 3, multicenter randomized, open-label	Adult SOT recipients	Vaccine response rate (>4 fold HAI titer increase)	Higher response with high-dose and MF59-adjuvanted vs standard dose	^106^
	2 doses standard IIV (5 weeks apart) vs 1 dose standard IIV (TRANSGIRPE 1−2)	Phase 3, randomized, controlled, open-label	Adult SOT recipients	Seroconversion and seroprotection at 10 weeks	Booster improved seroconversion and seroprotection	^79^
	High-dose IIV vs standard dose IIV	Double-blind randomized controlled trial	Adult SOT recipients	Seroconversion to >1 of 3 antigens at 4 weeks	Higher seroconversion and higher HAI GMTs in high dose group	^77^
	High dose IIV vs standard dose IIV	Phase I, Double-blind, randomized controlled trial	Pediatric SOT recipients	Safety and immunogenicity	No severe AEs or rejection, HD group demonstrated higher percentage of four-fold rise to H3N2 compared to SD	^78^

In addition to novel vaccines, the FDA also approved nirsevimab in 2023 for the prevention of RSV-associated lower respiratory tract disease in high-risk infants and young children. Nirsevimab is a long-acting monoclonal antibody targeting the prefusion conformation of the RSV fusion protein and has been shown to reduce medically attended RSV-associated disease in healthy infants.^46^ An additional study performed in 100 immunocompromised children (including solid organ transplant recipients) <24 months of age showed that nirsevimab was well tolerated and had serum concentrations suggestive of efficacy.^47^ Current guidance recommends nirsevimab for all infants <8 months born during or entering their first RSV season and 1 dose of nirsevimab for infants and children up to 19 months of age at increased risk for severe disease and entering their second RSV season.^48^ Currently, there are no ongoing clinical trials to assess the efficacy of nirsevimab in adult solid organ transplant recipients.

#### Treatment

Currently, there is no FDA approved antiviral therapy for RSV infection in lung transplant recipients. Ribavirin, a broad-spectrum nucleoside analogue with activity against both DNA and RNA viruses, has been used off-label for the treatment of RSV infection in this population, primarily based on limited clinical experience and extrapolated data. Existing guidelines for solid organ transplant recipients recommend aerosolized or oral ribavirin, with consideration for addition of corticosteroids and intravenous immunoglobulin (IVIG), for lung transplant recipients with upper or lower respiratory tract infection.^40^ However, the clinical efficacy of ribavirin remains uncertain.

Aerosolized ribavirin has been the traditional standard for RSV treatment in lung transplant recipients due to its direct delivery to the respiratory tract. Observational studies and clinical experience suggest that inhaled ribavirin may reduce progression from upper to lower respiratory tract infection and improve outcomes.^49^, ^50^ However, the evidence is limited to non-randomized studies, and no clear mortality benefit has been established in lung transplant recipients. In a large retrospective study, both oral and inhaled ribavirin showed no significant difference in adjusted mortality between the two groups; however, unadjusted 1-year mortality was higher in the inhaled group, likely reflecting greater baseline disease severity.^49^ A multicenter cohort study found that ribavirin (predominantly inhaled) was associated with a lower incidence of CLAD at 6 months post-infection (OR 0.24, 95% CI 0.10–0.59), and a significant improvement in post-infection FEV_1_ compared to no ribavirin.^51^ However, other studies have not found significant differences in lung function decline or allograft dysfunction at 3 months between ribavirin-treated and untreated patients.^38^ While inhaled ribavirin is generally well-tolerated, its use is limited by logistical challenges, high cost, and the need for prolonged (often 5–10 days) administration. Adverse effects are uncommon but may include bronchospasm, cough, and rarely, hemolytic anemia due to systemic absorption.

The cumbersome nature of aerosolized therapy has led many centers to consider oral ribavirin as a practical alternative, with several retrospective studies and systematic reviews demonstrating that oral ribavirin is generally well-tolerated and associated with similar clinical outcomes to inhaled ribavirin in terms of mortality, progression to lower respiratory tract infection, and preservation of lung function.^39^ In a large cohort, oral ribavirin was associated with recovery of FEV1 post infection and a low incidence of new onset BOS (2.6%) at 3 months post-infection.^52^

Direct comparisons between oral and inhaled ribavirin are limited to retrospective studies, which generally show no significant differences in clinical outcomes.^52^, ^53^, ^54^, ^55^, ^56^ Similarly, systematic reviews and meta-analyses have not demonstrated a clear mortality benefit for ribavirin (inhaled or oral) over supportive care in lung transplant recipients, but there is a trend toward reduced CLAD progression.^57^ The decision to initiate ribavirin therapy necessitates careful consideration of its potential toxicities, namely hemolytic anemia, against a backdrop of uncertain clinical efficacy.^52^

While ribavirin remains a widely utilized antiviral for the treatment of RSV in lung transplant recipients, limitations in its efficacy and its toxicity profile have prompted investigation of novel fusion inhibitors such as presatovir. Unfortunately, a randomized controlled trial of presatovir, published in 2023, failed to demonstrate clinical benefit; treatment did not significantly reduce nasal RSV viral load, improve symptoms, or preserve lung function in lung transplant recipients.^58^

Despite the lack of randomized trials demonstrating efficacy, the American Society of Transplantation Infectious Diseases Community of Practice supports the use of either oral or inhaled ribavirin in lung transplant recipients with upper or lower respiratory tract infection.^40^ However, adequately powered randomized controlled trials are still critically needed to establish the efficacy of ribavirin in this high-risk population.

### Influenza

#### Epidemiology and outcomes

Influenza infection can be particularly severe in lung transplant recipients, with relatively high rates of progression to lower respiratory tract infection.^59^, ^60^ Risk factors for severe disease include infection early (<3 months) after transplant, pre-existing BOS, use of antilymphocyte globulins, pneumonia, and bacterial and fungal co-infection.^40^, ^60^, ^61^ Influenza viruses are seasonal and circulate primarily during winter months. Rates of infection among lung transplant recipients can be variable depending on the circulating influenza strain and seasonable variability in influenza vaccine efficacy.^62^ A recently published prospective cohort study evaluating the incidence of respiratory viral infections in 3294 solid organ transplant recipients in Switzerland reported an incidence rate of 35.4 per 1000 patient-years in lung transplant recipients, compared to a rate of 13.1 per 1000 patient years in non-lung transplant recipients.^34^ Mortality can be substantial, with reported rates varying between 2–8%.^40^, ^60^, ^62^

Many studies have documented the complications of influenza in lung transplant recipients.^63^, ^64^, ^65^, ^66^, ^67^, ^68^, ^69^, ^70^ Among the most serious complications is the development of CLAD. Although several studies have suggested that some viruses, including influenza, were more commonly associated with the development of CLAD, other recent data using multiplex panels have not associated a particular respiratory virus with CLAD.^71^

#### Prevention

The most important means for prevention of influenza in the lung transplant population is immunization of the recipient and close contacts. In lung transplant recipients, vaccination with influenza A H1N1/09 vaccine was associated with a reduced incidence of subsequent influenza infection (1.3% vs 25% in unvaccinated patients).^72^ Furthermore, a prospective cohort including 577 adult and pediatric solid organ transplant patients with influenza infection showed that vaccination was associated with a significant reduction in disease severity, specifically a lower risk of pneumonia (OR 0.34) and ICU admission (OR 0.49).^59^ Influenza vaccines are available in several formulations, including standard dose tri-valent inactivated, high-dose tri-valent inactivated, and live-attenuated. Currently, only the inactivated influenza formulation is recommended in transplant recipients due to the theoretical risk of viral dissemination of the vaccine strain following live attenuated vaccine. The American Society of Transplantation (AST) guidelines recommend annual vaccination for all lung transplant candidates and recipients, starting as early as 1 month post-transplant.^73^

Immunogenicity of influenza vaccine in lung transplant recipients is significantly impaired compared to the general population, with immunogenic responses varying from 15–70%.^74^, ^75^, ^76^ Alternative strategies to overcome suboptimal immunogenicity in this population have been explored with variable outcomes. A recent randomized controlled trial of adult solid organ transplant recipients, including lung transplant recipients, demonstrated that a single dose of high dose influenza vaccine demonstrated significantly better immunogenicity than a single dose of standard dose influenza vaccine, with seroconversion to at least 1 of the 3 vaccine antigens in 78.6% in the high-dose group versus 55.8% in the standard dose group.^77^ Similar results were observed in a cohort of pediatric solid organ transplant recipients, though only two lung transplant recipients were included.^78^ Booster strategies in this population have also been investigated. A clinical trial of 499 solid organ transplant recipients reported improved seroprotection in all three vaccine strains in patients who received two doses of the standard dose influenza vaccine 5 weeks apart.^79^ Based on these data, AST guidance recommends that either high-dose or booster dosing in the same season may be preferred over standard dosing. A clinical trial to evaluate the immunogenicity and safety of two doses of high-dose influenza vaccine compared to two doses of standard dose influenza vaccine in lung allograft recipients is currently underway to address exiting knowledge gaps in optimal vaccination strategies in these patients (NCT 05215327).

Although influenza vaccination reduces the risk and severity of disease, for patients predicted to have a diminished response to vaccination (i.e., recent transplant, intense immunosuppression, and use of lymphocyte-depleting therapy for induction or treatment of rejection), seasonal antiviral prophylaxis with oseltamivir can be considered. A randomized trial of adult transplant recipients demonstrated ∼80% efficacy in reducing influenza incidence; however, this trial notably did not include any lung transplant recipients.^80^ In cases of contact with a patient with influenza, lung transplant recipients should be offered post-exposure prophylaxis with oseltamivir, regardless of vaccination status due to high risk for complications with infection.^40^

#### Treatment

The mainstays of influenza treatment are the neuraminidase inhibitors, oseltamivir, peramivir, and zanamivir. The influenza M2 protein inhibitors (amantadine and rimantadine) are approved for influenza A only but are no longer recommended for treatment of influenza due to the high incidence of antiviral resistance.^40^, ^81^ Early treatment with neuraminidase inhibitors, particularly oseltamivir, is recommended in lung transplant recipients. Multiple retrospective and prospective studies of solid organ transplant recipients have demonstrated that early initiation (within 48 h of symptom onset) of oseltamivir is associated with improved clinical outcomes and significantly reduced influenza-associated morbidity.^59^, ^60^, ^82^ In lung transplant recipients in particular, neuraminidase inhibitor therapy has been associated with reduced risk of the development of chronic lung allograft dysfunction.^82^ While early therapy initiated within 48 h of symptom onset is associated with better outcomes, therapy is still beneficial when initiated beyond 48 h, and current guidelines recommend that all lung transplant patients with influenza receive antiviral therapy, irrespective of duration of symptom onset.^40^ If clinical suspicion for influenza is high, given the suboptimal sensitivity of antigen-based rapid influenza diagnostic tests (generally ranging from 50–60%), nucleic acid amplification testing such as polymerase chain reaction (PCR) should be employed to facilitate appropriate use of antiviral therapy.^40^, ^83^

Baloxavir, a novel antiviral targeting the influenza cap-dependent endonuclease, was superior to both oseltamivir and placebo in decreasing viral loads among healthy outpatients and may be a promising therapy for otherwise healthy individuals.^84^ However, the clinical efficacy and safety in solid organ transplant recipients have not been well established due to limited representation of this population in current studies. Due to risk of resistance and limited clinical data, baloxavir is not currently recommended for use in lung transplant recipients.^85^, ^86^

### Human metapneumovirus (hMPV)

#### Epidemiology and outcomes

HMPV has been identified in lung transplant recipients with varying incidence rates. A five-year prospective study of 98 lung transplant recipients detected hMPV in 7.7% of patients. In contrast, a more recent retrospective analysis of respiratory viral infections over a 10-year period found hMPV in 33% (46/139) of lung transplant recipients, all of whom were symptomatic. The majority of cases occurred between December and March, with a lower incidence observed during the summer months. Notably, 65% of affected patients developed severe infection.

Similar to RSV, hMPV has a propensity to progress to lower respiratory tract disease, leading to increased morbidity and mortality in lung transplant recipients. A recent systematic review and meta-analysis of respiratory viral infections in this population estimated a 30-day mortality rate of 2% for hMPV. Given its association with lower respiratory tract disease, a systematic literature search was conducted to identify cases of hMPV infection and allograft rejection within six months of initial diagnosis. This study included 1007 lung transplant recipients, 57 of whom had isolated hMPV infection without co-infection with other pathogens. Among these patients, 35% developed acute cellular rejection within three months, and 9.4% subsequently developed CLAD. Similarly, the aforementioned meta-analysis reported a high pooled CLAD incidence of 12% following hMPV infection. A retrospective study of all lung transplant recipients at Johns Hopkins Hospital from July 2010 to June 2019 found that 35.5% of hMPV-infected patients experienced CLAD progression within one year. The risk of CLAD development was more pronounced in patients with lower respiratory tract disease and those who failed to achieve early lung function recovery. Large, prospective multicenter studies are needed to better define the risk factors and clinical impact of HMPV in this population.

#### Prevention

As with other RVIs, interventions at a health care system level can decrease the incidence of hMPV disease; these include appropriate use of respiratory, contact, and droplet precautions, and hand hygiene. Unfortunately, no licensed vaccines or antiviral therapies are available for the prevention of hMPV, although messenger RNA (mRNA) vaccines are currently under investigation. A recent phase I clinical trial evaluated the safety and humoral immunogenicity of a novel mRNA-based hMPV and parainfluenza virus 3 combination vaccine in healthy adults. A single dose increased neutralization titers against hMPV, which persisted one year after vaccination.^87^ Several ongoing clinical trials are evaluating hMPV vaccine candidates (NCT05743881, NCT06686654); however, none currently include transplant recipients, highlighting a critical gap in research for this high-risk population.

#### Treatment

There are currently no approved antiviral therapies for the treatment of hMPV. Ribavirin has shown in vitro activity against hMPV. However, there are no controlled studies or data from large retrospective studies demonstrating clinical efficacy, and its role in treating infections with hMPV is unclear. The aforementioned recent systematic review of RSV, hMPV, and parainfluenza virus infections in lung transplant recipients by De Zwart et al. included studies reporting treatment with ribavirin, either alone or in combination with IVIG. Data on effectiveness of ribavirin were mostly available from studies not specifically designed or powered to detect effectiveness, and outcomes were conflicting. One prospective study compared lung transplant recipients treated with oral ribavirin to those not treated with ribavirin and noted new onset CLAD at 6 months in 5% of the treated group versus 24% of the non-treated group.^39^

There is currently no consensus supporting the routine use of ribavirin for the treatment of hMPV infection in lung transplant recipients. Current guidelines suggest that a ribavirin with or without IVIG and corticosteroids may be considered for the treatment of lower respiratory tract disease, although this recommendation remains weak in the absence of robust clinical trial data supporting its use.^40^ Emphasis is placed on supportive care as the mainstay or treatment, with ribavirin as a consideration in severe cases and on a case-by-case basis. This lack of a uniform recommendation reflects both the limited and inconsistent efficacy data and the potential for clinically significant adverse effects, particularly hematologic toxicity. Thus, the risk-benefit profile of ribavirin in this setting remains uncertain, and its use should be individualized, weighing the severity of infection against the potential for harm.

### Adenovirus

#### Epidemiology and outcomes

Adenovirus infection in lung transplant recipients can result in significant graft dysfunction and lead to high mortality.^88^ Unlike some respiratory viruses, adenovirus circulates year-round without a distinct seasonal pattern.^89^ In the solid-organ transplant population, adenovirus can be acquired *de novo*, through reactivation of latent infection, or be transmitted through the donor organ. The overall incidence of adenovirus infection in lung transplant recipients remains poorly defined, though it appears to be more frequently detected in pediatric recipients. Reported incidence rates vary substantially, ranging from 7–50% in pediatric lung transplant recipients and 6–22.5% in adult recipients.^89^

Adenovirus infection in lung transplant recipients presents with a wide clinical spectrum, ranging from asymptomatic viremia to severe disseminated disease. Asymptomatic shedding of adenovirus occurs when adenovirus is detected in respiratory secretions, blood, or other specimens in the absence of clinical symptoms. The AST recommends against surveillance testing for adenovirus in asymptomatic recipients, as viremia or shedding is common and typically self-limited, with no impact on graft function or outcomes in the absence of symptoms or organ involvement.^89^ When symptomatic, clinically relevant adenovirus disease in lung transplant recipients frequently presents with an acute flu-like illness or pneumonia, which can progress to diffuse alveolar damage, BOS, interstitial fibrosis, or bronchiectasis. Lung transplant recipients appear to be particularly susceptible to necrotizing pneumonitis, which can lead to significant graft dysfunction.^89^, ^90^ Risk factors for progression from asymptomatic infection to disease include infection early after transplantation, isolation of adenovirus from multiple sites, high viral load, and intensification of immunosuppression.^89^ Several reports have linked adenoviral infection of the allograft to both acute and chronic rejection and graft dysfunction.^88^ In a small prospective study of 16 predominantly pediatric lung transplant recipients, adenoviral respiratory tract disease occurred in 8 patients and was significantly associated with the subsequent development of BOS. In contrast, a more recent prospective study of 80 adult lung transplant recipients reported 18 of 80 patients (22.5%) developed adenovirus viremia in the first year of transplantation with no detrimental effect of pulmonary function. These isolated episodes of low-level viremia were self-limited and did not trigger episodes of acute rejection.^91^

#### Treatment

The use of antivirals in the treatment of adenovirus infection is not supported by randomized clinical trials, and there are currently no antiviral agents with FDA approval for the treatment of adenovirus. As for most viral infections in lung transplant recipients, supportive care and reduction of immunosuppression is recommended if clinically feasible. Cidofovir, a nucleotide analogue with in vitro activity against adenovirus, is the most frequently used antiviral despite its nephrotoxicity and is standard practice for treatment of severe or disseminated adenovirus in most transplant centers.^89^, ^92^ The evidence base for cidofovir is limited by small sample sizes, lack of control groups, and heterogeneity in patient populations and adjunctive therapies. Some case reports and small case series suggest that cidofovir may improve outcomes, particularly when administered early in the course of disease.^93^ A pediatric lung transplant case series demonstrated that cidofovir for the treatment of adenovirus pneumonia was associated with clinical improvement and viral clearance, especially when combined with renal protection strategies (probenecid, intravenous hydration). In the cited series, three of the four infected children survived with this regimen and maintained normal renal function. Additional retrospective studies in solid organ transplant recipients support the use of cidofovir for symptom resolution, but these studies included limited lung transplant recipients.^94^, ^95^ Typically, one of two regimens of cidofovir are used: 5mg/kg once every 7 days, or 1mg/kg IV three times a week in conjunction with hydration and probenecid to prevent nephrotoxicity.^89^ Despite limited data, cidofovir remains the antiviral of choice for severe adenovirus disease in lung transplant recipients, with dosing and duration individualized based on clinical response and tolerability.

Brincidofovir, a prodrug of cidofovir with decreased nephrotoxicity, has shown promise in the stem cell transplant population, but remains investigational for the solid organ transplant population.^96^, ^97^ A small case series of four pediatric solid organ transplant recipients with severe adenovirus infection demonstrated successful treatment with brincidofovir; however, no lung transplant recipients were included.^98^ Adjunctive use of IVssIG has been reported in several cases of severe disease. However, the benefits of this therapy remain unclear.

## Management considerations

### Future directions

Despite growing recognition of the clinical importance of respiratory viral infections in lung transplant recipients, significant gaps remain in evidence-based management, preventive strategies, and long-term outcomes. Large, prospective, multicenter studies are needed to define optimal management approaches and clinical effectiveness and safety of antiviral therapies. Development and inclusion of transplant recipients in clinical trials of novel antiviral agents and vaccines, including those targeting RSV, hMPV, and emerging respiratory pathogens, is urgently needed. Additionally, a deeper understanding of the relationship between RVIs and CLAD remains a critical priority. Elucidating the mechanisms by which viral infections contribute to graft injury and fibrosis could inform both preventive strategies and novel therapeutic targets aimed at preserving long-term graft function.

## Declaration of Competing Interest

The authors declare the following financial interests/personal relationships which may be considered as potential competing interests: Lara Danziger-Isakov reports a relationship with Merck & Co Inc that includes: consulting or advisory and funding grants. Lara Danziger-Isakov reports a relationship with AiCuris Anti-Infective Cures GmbH that includes: funding grants. Lara Danziger-Isakov reports a relationship with Takeda Pharmaceuticals USA Inc that includes: funding grants. Lara Danziger-Isakov reports a relationship with Ansun BioPharma Inc that includes: funding grants. Lara Danziger-Isakov reports a relationship with Pfizer Inc that includes: funding grants. If there are other authors, they declare that they have no known competing financial interests or personal relationships that could have appeared to influence the work reported in this paper.
